# Lateral mass screw fixation in cervical spine injury

**DOI:** 10.12669/pjms.336.12947

**Published:** 2017

**Authors:** Lal Rehman, Iram Bukhari, Ali Afzal, Raza Rizvi

**Affiliations:** 1 Dr. Lal Rehman, FCPS. Department of Neurosurgery, Jinnah Postgraduate Medical Center, Karachi, Pakistan; 2 Dr. Iram Bukhari, FCPS. Department of Neurosurgery, Jinnah Postgraduate Medical Center, Karachi, Pakistan; 3 Dr. Ali Afzal, MBBS. Department of Neurosurgery, Jinnah Postgraduate Medical Center, Karachi, Pakistan; 4 Dr. Raza Rizvi, MS. Department of Neurosurgery, Jinnah Postgraduate Medical Center, Karachi, Pakistan

**Keywords:** Cervical Injury, Lateral Mass Screw

## Abstract

**Objective:**

To determine clinical outcome in patients with cervical injury after lateral mass screws fixation in a tertiary care hospital.

**Methods:**

This study included 88 patients, with cervical injury confirmed radiologically. Patients <12 or >70 years, with traumatic discs, cord compression without subluxation and previously operated on cervical spine were excluded from this study. All patients underwent fixation with lateral mass screws through posterior approach under fluoroscopic guidance. Frankel grading was used to assess the clinical status of these patients pre-operatively & post-operatively.

**Results:**

There were 60(68.18%) males and 28(31.8%) females. The ages varied from 18 to 55 years with a mean of 32 yrs ± 8 yrs. The most common level of injury was C5-C6 in 46(52%) patients. According to Frankel grading system, 35 (39.8%) patients were placed in Grade A, 15(17.05%) in Grade B, 22(25%) in Grade C, 12 (13.6%) in Grade D, four (4.5%) in Grade E on admission. Postoperatively, 16 (18.2%) patients were placed in Grade A, 23 (26.1%) in Grade B, eight (9.1%) in Grade C, nine (10.2%) in Grade D and 26(29.6%) patients in Grade E with an overall improvement in neurological function in 51(58%) and power in 37(42%) patients. The major complications encountered were respiratory infections in 10(11.36%) and wound infection in four (4.5%) while eight (9.1%) patients expired.

**Conclusion:**

Lateral mass screws technique is a safe and effective method for cervical fixation after proper reduction.

## INTRODUCTION

Cervical spine injury is very common; the reason ascribed being an increased mobility of cervical spine, making it susceptible to trauma & variety of degenerative diseases.^1^ Extreme difficulty is encountered in performing surgery in this region because of close proximity to many vital structures. Cervical cord compression leads to weakness in all four limbs with MRI being the investigation of choice. CT with 3D reconstruction helps in surgical planning. Surgery can be performed both from anterior as well as posterior sides. Lateral mass screw fixation (LSF) with plates or rods has become the standard method for posterior cervical spine fixation and stability.^2^

After development of the polyaxial screw-rod system, cervical fixation surgery has now become easier to perform. Most surgeons now believe the LSF techniques are optimum methods for reconstructing the stability of the cervical spine, following decompressive surgery.^3^,^4^ Despite its ease of application and better biomechanical stability, when compared with other techniques, the main risk remains that of violating the spinal nerve root, vertebral artery, and/or facet joint.^5^,^6^ The rationale of this study was to describe the safety profile and effectiveness of such system when used in stabilizing the cervical spine.

## METHODS

This descriptive study was conducted at the department of neurosurgery at JPMC from 1^st^ December, 2012 to 31^st^ December, 2015 after institutional review board approval. This study included 88 patients, with cervical injury confirmed radiologically exhibiting subluxation that was not reduced after cervical traction. Inclusion criteria were subaxial spine injury with subluxation and unstable subaxial injury. Patients <12 or >70 years, with traumatic discs, cord compression without subluxation and previously operated on cervical spine were excluded from this study.

The average time from injury to intervention was five days and up to a maximum of 15 days. All injuries were confirmed radiologically by X-rays, CT scans with 3D reconstruction & MRI of cervical spine. Cervical traction & collar were given. They were operated and underwent fixation with lateral mass screws through posterior approach under fluoroscopic guidance in prone position. After exposure and separation of cervical muscles, the screw was passed in a trajectory through lateral mass1 mm medial and 1 mm inferior at midpoint of lateral mass with 20 degree upward and lateral direction in order to prevent neurovascular injury. Placement was confirmed by fluoroscopy and fixations were done with rods. After reduction bone chips were placed on facet joints for fusion. Postoperative radiology was done immediately after surgery and at three and six months after surgery.

Data was collected with the help of proformas and Frankel grading system was used to assess the clinical status of these patients pre operatively &improvement at six months after operation as shown in Table-I. Any complication or mortality was noted. SPSS version 22 was used for statistical analysis. Categorical variables, such as gender, level of injury, Frankel grades pre-operatively and post-operatively were expressed in frequency and percentage, whereas continuous or quantitative variables such as patient’s age was expressed in mean ±SD with range. Chi square test was applied post stratification and P-value of ≤0.05 was taken as significant.

**Table-I:** Frankel Grading System.

Provides an assessment of spinal cord function as follows:	
Grade A	Complete neurological injury - No motor or sensory function detected below level of lesion
Grade B	Preserved sensation only - No motor function detected below level of lesion, some sensory function below level of lesion preserved
Grade C	Preserved motor, nonfunctional - Some voluntary motor function preserved below level of lesion but too weak to serve any useful purpose, sensation may or may not be preserved
Grade D	Preserved motor, functional - Functionally useful voluntary motor function below level of injury is preserved
Grade E	Normal motor function - Normal motor and sensory function below level of lesion, abnormal reflexes may persist

## RESULTS

**Fig 1:**
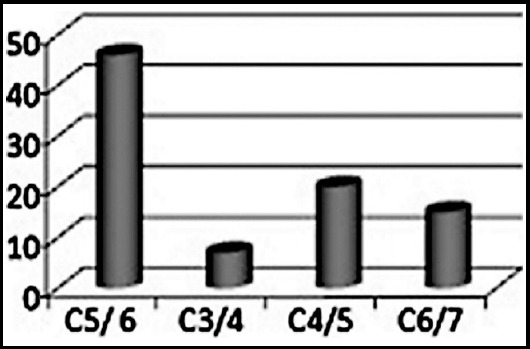
Level of injury in patients with Cervical Trauma (n= 88).

This study included 88 patients out of which 60(68.18%) were male compared to 28 (31.8%) females 51 (68.75%). The age varied from 18 to 55 years with a mean of 32 yrs ± 8 yrs. The most common level of injury was C5-C6 in 46(52%) patients (Fig.1). According to Frankel grading system, 35(39.8%) patients were placed in Grade A, 15(17.05%) in Grade B, 22(25%) in Grade C, 12 (13.6%) in Grade D, four(4.5%) in Grade E. Postoperatively, 16 (18.2%) patients were placed in Grade A, 23 (26.1%) in Grade B, eight(9.1%) in Grade C, nine(10.2%) in Grade D and 26(29.6%) patients in Grade E as shown in Table-II with an overall improvement in neurological function in 51(58%)[p-value 0.001]and power in 37(42%) patients, as shown in Table-III. All four patients in Grade E remained same post operatively. The complications in decreasing order were respiratory infections in 10(11.36%), wound infection in four (4.5%), root injury in three (3.4%) and vertebral artery injury in one (1.1%) patient while eight (9.1%) patients expired. All patients with infection were managed by high dose antibiotics & daily dressing. None of these patients required any reoperation. Eight patients (9.1%) expired. There were no procedural related deaths. Four patients expired due to respiratory compromise and four secondary to pulmonary embolism.

**Table-II:** Pre & Post- operative Frankel Grades (at 3 months) in patients (n=88).

*Pre Operative*		*Post Operative*						

*Frankel Grade*		*A*	*B*	*C*	*D*	*E*	*Exp*	*Total*
A	35	16	14	2			3	35
B	15		9	4			2	15
C	22			2	9	10	1	22
D	12					12		12
E	4					4		4

Total	88	16	23	8	9	26	6	88

**Table-III:** Group wise outcome comparison.

		*Post Operative*	

*Frankel Grades*	*Pre Operative (n=88)*	*Neurological Improvement Frankel Grade B & above P value< 0.001*	*Improvement in power Frankel Grade D & E P value<0.001*
Group-1 (A+B)	50	20	6
Group-2(C+D)	34	31	31

Total	82	51	37

## DISCUSSION

Traumatic spinal cord injury is common with most tragic outcomes in the cervical spine.^7^ Extreme difficulties are encountered during surgical intervention in these patients due to complex anatomical nature of this region and association with vital structures. Recent studies show that early decompression results in a more favorable outcome.^8^ Different techniques both from anterior and posterior approaches are used for the decompression and stabilization of the cervical spine. Of these, lateral mass screw fixation has become the method of choice among other posterior cervical fixation techniques whenever the posterior elements are absent or compromised.^9^

Small clinical series and biomechanical data support their role as a substitute for other posterior stabilization techniques; however, the application of transarticular facet screws in the subaxial cervical spine has not been widely adopted, possibly because of surgeon unfamiliarity with the trajectory.^10^ After initial description by Roy-Camille, several techniques for LSF have been described^11^,^12^, subsequently popularized by Louis and Magerl and more recently by Anderson and Ebraheim.^13^

Anatomically, the lateral or articular mass consists of the superior and inferior articular facets and is anterolateral to lamina. We used midpoint of lateral mass as a reference point and screw was passed in a trajectory through lateral mass1 mm medial and 1 mm inferior at midpoint in 20 degree upward and lateral direction under fluoroscopic guidance, in order to prevent neurovascular injury. Since the trajectory is directed away from the spinal cord, this technique has a lower risk of injuring the spinal cord as suggested by Magerl^13^ and widely followed in clinical studies like Wang et al.^14^

Neurologic injury can also be caused by insertion of long screws leading to a disruption of the ventral cortex of the lateral mass. The oblique antero-posterior (OAP) diameter of the articular pillar is representative of the screw length by Magerl method with average OAP diameter reported from 10.8 mm to 20.3 mm with a mean of 14.9 mm ± 1.8 as reported by Sangari et al.^15^ similar to our study where we used screw between12to 14 mm. Even, aiming the screw anteromedially rather than anterolaterally can lead to penetration of the transverse foramen and thus vertebral artery injury.

Other complications include screw loosening and pull out. Despite these possible complications, lateral mass screws have an excellent safety profile as observed in our study as none of the patients developed neurological deterioration. Similar to our complication rate, Katonis et al.^16^ found no cases of vertebral artery, exiting nerve, or spinal cord injury attributable to screw placement. Graham and Roche^17^ claim that screw positioning is the main factor leading to this complication. Though Roche established fluoroscopy is not essential, we think that using the fluoroscope is essential as many patients have altered anatomy and using this not only improves safety profile but also the accuracy of screw placement. Thus, confirmation of a trajectory under fluoroscopic guidance is important for safety of this technique.

In this study, improvement was seen in motor & sensory function as assessed by Frankel Grading System at six months after stabilization with LMS placement with an statistically significant improvement in neurological function in 51(58%) p value 0.001 and power in 37(42%) patients, p value< 0.001(Table-III)as shown by Yehya et al.^18^ This is based on Frankel grading where overall improvement implies sensory and motor function both whereas power denotes motor function alone. None of the patients deteriorated after surgical intervention. Other studies,^19^ have shown that lateral mass screw-rod fixation followed by fusion shows promise as an effective and biomechanically sound way of treatment in properly selected cervical injury cases.

Although, safe and reliable, it is difficult to use in patients with abnormal cervical anatomy as it may lead to injury of the spinal nerves or the vertebral arteries during screw insertion^20^ which is why we used 3D CT scan for measurement of size and shape of lateral mass preoperatively in all cases. Therefore, it is recommended that surgeons using this technique should have familiarity and intimate knowledge of cervical anatomy with adequate preoperative evaluation for each patient, with the final selection based on individual case requirements and anatomical limitations.

## CONCLUSION

Lateral mass screws are a safe and effective method for cervical fixation after proper reduction that not only stabilizes the cervical spine but results in satisfactory functional recovery of patients.
